# Exploring Infant Caregivers' Provision of Modified Formulas: Potential Demographic Differences and Reasons for Provisions

**DOI:** 10.3389/fnut.2022.867932

**Published:** 2022-05-18

**Authors:** Haley Gershman, Maria J. Romo-Palafox, Tassneem Rajeh, Frances Fleming-Milici, Jennifer L. Harris

**Affiliations:** ^1^Rudd Center for Food Policy and Health, University of Connecticut, Hartford, CT, United States; ^2^Department of Nutrition and Dietetics, Doisy College of Health Sciences, Saint Louis University, St. Louis, MO, United States

**Keywords:** infants, health, infant formula, regulations and policy, labelling requirements

## Abstract

**Background:**

Formula brands have modified the ingredients in standard infant formulas and extensively market modified formulas, claiming benefits for infants that are not supported by scientific evidence. This exploratory study examined the proportion of infant caregivers who reported serving modified formula, demographic differences, and reasons for providing them.

**Methods:**

This is a cross-sectional online survey of US caregivers of infants (6–11 months) who provided formula in the past month (*N* = 436). Participants reported the type of formula served most often and agreement with potential reasons for provision. Logistic regression assessed the odds of serving modified formula by demographic characteristics. MANOVA examined differences in agreement with purchase reasons between caregivers by the type of formula provided.

**Results:**

Approximately one-half (47%) of participants reported serving modified formula most often; sensitive and organic/non-GMO were the most common types provided. Caregivers in the middle-income group were most likely to serve modified formulas, but the provision did not differ by other demographic characteristics. Agreement with reasons for providing was highest for “pediatricians recommend” and “benefits my child” (*M* = 4.2 out of 5). Agreement with “my pediatrician prescribed” and “natural ingredients” was significantly higher for modified vs. standard formula providers.

**Conclusion:**

Widespread provision of modified formula by infant caregivers raises concerns due to its higher cost and the lack of scientific evidence supporting benefits for babies. These findings suggest that regulations limiting unsubstantiated formula claims and restrictions on misleading marketing to consumers are necessary. Additional research is needed to understand pediatricians' perceptions of modified formulas and reasons for recommending them to patients.

## Introduction

The World Health Organization (WHO), the American Academy of Pediatrics (AAP), and other health organizations recognize breast milk as the best source of nutrition for infants (1–3). In recent years, rates of breastfeeding have increased (4). Yet only one-quarter of US infants born in 2018 were exclusively breastfed for 6 months, and there are disparities in breastfeeding rates based on race, maternal education, age, and family income (4). For caregivers and infants who cannot breastfeed, standard infant formula provides an acceptable alternative, which according to the American Academy of Family Physicians should contain lactose as the carbohydrate source and cow's milk protein as the protein source (5). The US Food and Drug Administration (FDA) also specifies nutrient requirements for infant formulas, including required levels of protein, fat, carbohydrates, vitamins, and minerals per 100 calories (6).

However, in the face of increased breastfeeding rates and a competitive infant formula market, many formula brands have modified the ingredients in their standard infant formulas to differentiate their products and charge a premium price (7). These ingredient modifications include reduced-lactose, hydrolyzed or soy proteins, and organic or non-GMO ingredients, and the addition of ingredients, such as DHA, prebiotics, or probiotics (7). Companies then market these modified formulas to consumers with claims that suggest that they should reduce common infant feeding problems, such as fussiness or gas, or provide other benefits to babies, including brain development and growth (7–9). Modified formulas contributed 79% of all infant formula consumer advertising spending in 2015 (10). Modified formula products also comprise an increasing proportion of formula sales (11). Although total infant formula volume sales declined by 7% from 2006 to 2015, dollar sales increased by 24% due to the higher price-per-ounce of modified formulas compared with standard formulas (12).

In addition to their higher price, child health experts have raised concerns about the potentially misleading claims that are used to promote infant formulas to consumers (7–9, 13–16). Common claims, such as “closest to breast milk,” “#1 pediatrician recommended,” and promises of the brain, neural, and/or gastrointestinal benefits, imply that some formulas are better for infants than standard formulas (14, 15). However, the US Food and Drug Administration (FDA) does not require companies to support these types of structure/function claims (i.e., claims that link product ingredients with bodily functions) with high-quality scientific evidence (17). A systematic review of 307 intervention trials that compared two or more formula products found that just 4% had a low risk of bias and 80% had a high risk due to inappropriate exclusions and selective results reporting (18). Moreover, these types of claims lead parents to infer that modified formulas even provide benefits for infants over breastfeeding (14, 15).

Despite these concerns, limited research has examined the extent of caregivers' provision of modified vs. standard formulas or their reasons for choosing these products. The purpose of this study was to examine the proportion of caregivers who reported serving modified formulas to their infants most often, understand demographic differences in their provision, and explore caregivers' reasons for providing modified formulas.

## Methods

This exploratory analysis utilized data from a large cross-sectional online survey of US caregivers of infants and toddlers conducted in 2017. Details of the survey have previously been described (15, 19). The survey examined a broad range of caregiver behaviors and beliefs about feeding their child. Participants answered questions about one infant or toddler (6–36 months old). This study reports results for caregivers of infants (6–11 months) and the formula products they provided their child most often. The University of Connecticut Institutional Review Board approved this study.

### Participants

Two national online survey panels invited their eligible panel members to participate in this study, namely, Innovate Market Research (a large national panel) and Offerwise (Hispanic household panel). Panelists from Offerwise could view and complete each question in Spanish or English. To allow for meaningful comparisons between racial/ethnic groups, researchers set quotas for Black non-Hispanic and Asian participants, as well as Hispanic participants of higher and lower acculturation levels. Additional quotas ensured equal numbers of children by age group (6–11 months, 12–24, and 25–36 months). Caregivers were excluded if they did not have at least one child aged 6–36 months, decision-making responsibility regarding what to feed their child, or they had a child with a health condition that requires a special diet (e.g., lactose intolerance).

### Measures

#### Caregiver Demographics

Participants reported their racial and ethnic background, highest level of education, and annual household income. Researchers coded racial/ethnic groups as follows: non-Hispanic White, non-Hispanic Black, Hispanic, Asian, and mixed/other. Additionally, Hispanic participants answered the Short Acculturation Scale for Hispanics (SASH), a validated tool that assesses language preference (20). As per SASH methodology, Hispanic participants were classified as less acculturated (score <3.0) or more acculturated. Researchers coded caregivers into low (high school or GED), middle (some college or 2-year college), and high (college graduate or higher) education groups and low (< $40,000), middle ($40,000–$74,999), and higher ($75,000 or more) income groups.

#### Formula Product Served

Participants first indicated if they had served formula to their child (6–11 months) in the past month. Those who answered “yes” then viewed a list of formula brand logos to select the brand they served most often in the past month. The list included the logos of six of the most marketed formula brands, based on advertising spending in 2015 (10). Then, participants saw a list of product images and names, including standard and modified formula products offered by the selected brand. Participants chose the product they had served most often in the past month to their child. Participants could also select “other” and include a product name if the product they served most often was not on the list. Specialty formulas for infants with food allergies or other medical conditions were not shown.

#### Product Categories

Researchers categorized the formula product caregivers as standard or modified (refer to Supplemental Material for a list of formulas by category). Modified formula product names usually contained the words “sensitive” (or “gentle”), “organic,” “non-GMO,” “supplementing,” or “soy.” Based on the product name, modified formula products were further coded as sensitive, organic/non-GMO, supplemental, or soy. If the product name did not provide enough information to categorize the formula, the researchers accessed the product package online and categorized the product based on the information on the package. Any product that could not be categorized was designated as “other,” and these participants were not included in the final analysis.

#### Purchase Reasons

Participants then were shown the picture and name of the product they had indicated they served most often in the past month and asked to rate their level of agreement with 12 possible reasons for purchasing it using a 5-point Likert scale (“strongly disagree” to “strongly agree”). The question asked, “I purchased this product because:” followed by reasons that reflect common benefit claims in infant formula marketing (“It has ingredients that will help my child grow,” “It has ingredients that will help my child's brain,” “It is easiest on my child's tummy,” and “Pediatricians recommend it”); product ingredient claims (“It is organic or natural,” “It does not contain added sugar,” and “It does not contain GMOs”); and other potential reasons for providing a specific formula or formulas in general (“It is convenient,” “It is affordable,” “It is healthy,” “It is the best option for a child this age,” and “My pediatrician prescribed it” [referring to their own pediatrician]). We included two different purchase reasons that refer to pediatricians. The first one, “Pediatricians recommend it,” assesses a common benefit claim on infant formula packaging that refers to pediatricians in general (10). The second one, “My pediatrician prescribed it” assessed the participant's personal experience with discussing formula options with their child's pediatrician.

#### Statistical Analysis

We reported descriptive statistics for participants who reported serving any formula to their child (6–11 months) in the past month, including socio-demographic characteristics and the type of formula served most often. A logistic regression model assessed the odds of serving a modified formula product by caregiver demographic characteristics (race/ethnicity, education, and income). Researchers used exploratory factor analysis to identify factors that best explained these reasons. Two factors emerged. One factor included reasons related to benefits for infants (healthy, best option, helps child grow, helps child's brain, and easiest on tummy) (Cronbach's α = 0.87). The other factor included reasons related to natural ingredients (organic/natural, no added sugar, and non-GMO) (Cronbach's α = 0.72). Four additional reasons had factor loadings below 0.7 and were included as separate variables: Affordable, convenient, pediatricians recommend, and my pediatrician prescribed. Multivariate analysis of variance (MANOVA) was used to compare differences in purchase reasons between caregivers who provided standard vs. modified formulas. All analyses were conducted using Statistical Analysis System software (SAS version 9.4).

## Results

After exclusions and incomplete responses, 555 caregivers of infants (6–11 months) completed the survey. In addition, 119 caregivers (24%) were excluded because they did not serve any formula in the past month. The final sample (*N* = 436) was diverse in race/ethnicity, with 27% non-Hispanic White, 33% non-Hispanic Black, and 24% Hispanic participants (Table 1). More than 40% of participants reported an annual household income under $40,000 (44%), and 55% reported having less than a college education. Of caregivers who reported serving any formula in the past month, approximately one-half (47%) reported serving standard formula most often. Of the 47% of caregivers who reported serving a modified formula most often, sensitive was the most common type served (37%), followed by organic/non-GMO (32%), supplemental (19%), and soy (12%). Another 26 caregivers reported serving “other” formulas and were excluded from the remaining analyses.

**Table 1:** Participant characteristics (*N* = 436).

**Socio-demographic characteristics**	***N***		**%**	
Caregiver race/ethnicity				
White non-Hispanic	119		27	
Black non-Hispanic	145		33	
Hispanic: more acculturated	50		11	
Hispanic: less acculturated	56		13	
Asian	58		13	
Mixed/other	8		2	
Household income^*^				
Under $40,000	190		44	
$40,000–$74,999	145		33	
$75,000 or more	97		22	
Caregiver education				
High school or GED	71		16	
Some college or 2-year college	170		39	
College graduate or higher	195		45	
Formula type served most often				
Standard	204		47	
Modified	206		47	
Sensitive		77		37
Organic/non-GMO		66		32
Supplemental		39		19
Soy		24		12
Other	26		6	

* *Does not total 100% due to missing data*.

**Figure 1:**
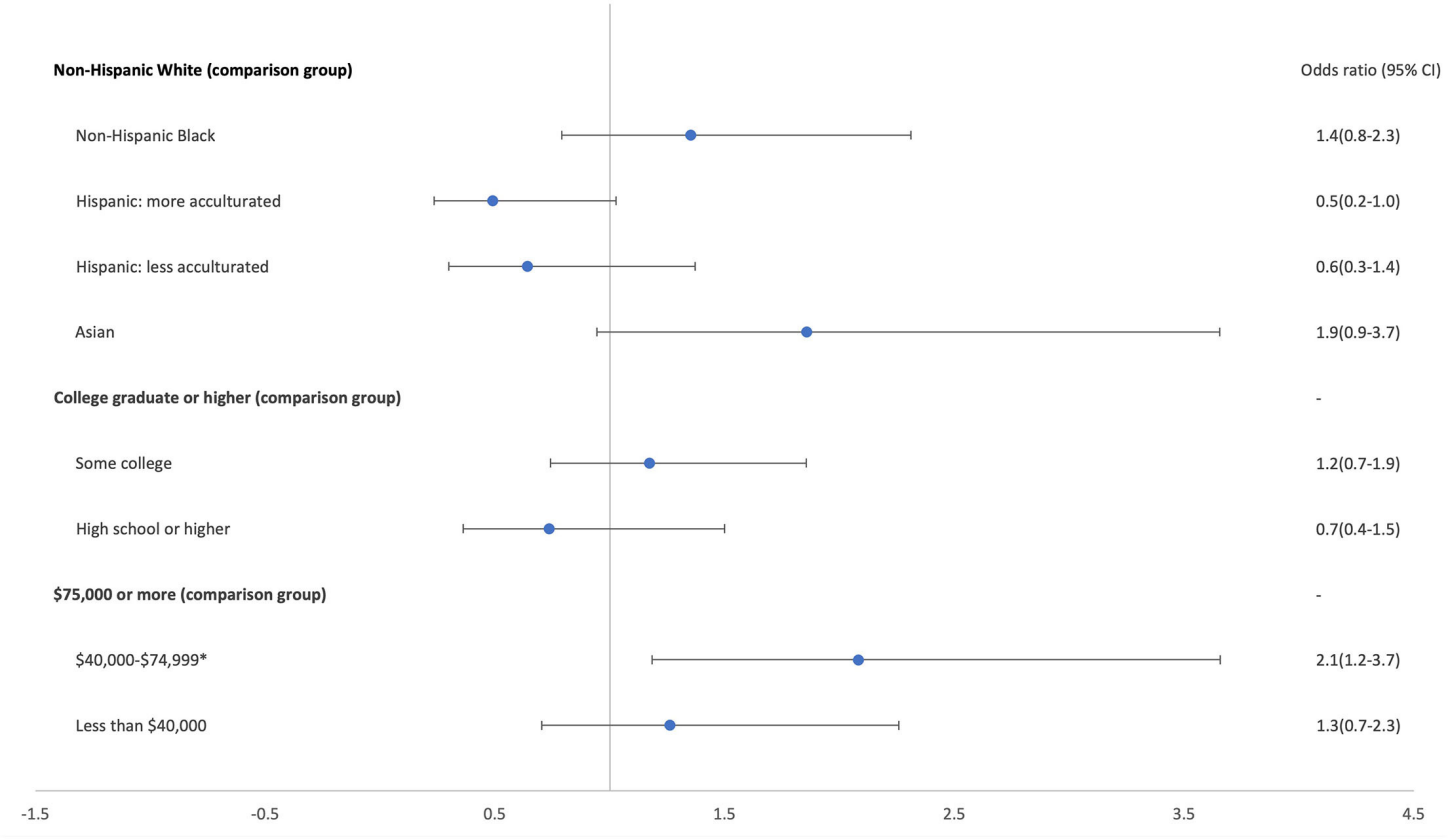
Odds of serving modified formula by caregiver demographics (*n* = 401) (*p < 0.001). Excludes 9 participants who reported mixed/other race and/or did not provide complete demographic information.

Figure 1 presents the results of the logistic regression model to examine associations between caregiver demographics and modified formula provision. There were no differences in odds of providing modified formula by caregivers' race/ethnicity or education level or between higher and low-income caregivers. However, caregivers in the middle-income group had approximately double the odds of serving modified formula compared with those in the highest income group.

Table 2 presents caregivers' agreement with different reasons for providing the formula product they served most often, including differences between those who served standard vs. modified formulas. The agreement was high for all potential reasons for providing both standard and modified formula products (*M* = 3.6 or higher out of 5). Mean agreement with pediatricians recommend it, it has benefits for infants, and it is convenient exceeded 4.0 for both types. Significant differences were found between caregivers who provided modified vs. standard formulas in agreement that “my pediatrician prescribed it” and natural ingredients. Despite their differences in price and ingredients, affordability ratings were the same for both types of formula.

**Table 2:** Agreement with purchase reasons by formula product category served (*N* = 410).

**Purchase reasons**	**Standard formula (*n* = 204)**		**Modified formula (*n* = 206)**	
	**Regressed mean**	**95% confidence limits**	**Regressed mean**	**95% confidence limits**
Pediatricians recommend	4.19	(4.06–4.32)	4.17	(4.04–4.31)
Benefits for infants^a^	4.21	(4.10–4.32)	4.16	(4.05–4.26)
Convenient	4.14	(4.00–4.29)	4.08	(3.93–4.22)
My pediatrician prescribed^*^	3.63	(3.46–3.80)	3.90	(3.73–4.07)
Natural ingredients^*^^b^	3.70	(3.59–3.81)	3.86	(3.75–3.98)
Affordable	3.87	(3.72–4.02)	3.85	(3.71–4.00)

a *Factor includes healthy, best option, helps child grow, helps child's brain, and easiest on the tummy.*

b *Factor includes organic/natural, no added sugar, and non-GMO.*

* *p <0.05*.

## Discussion

To our knowledge, this study is the first to document the widespread provision of modified formula, with almost one-half of caregivers who served formula to their infant in the past month reporting providing a modified formula most often. This finding raises concerns given the higher cost of modified formulas and the lack of evidence supporting claims that they provide any benefits for infants (5, 7, 9, 13, 18). Of additional concern, caregivers in low-income households were equally likely to provide these higher-priced modified formulas compared with caregivers in higher-income households, and caregivers in mid-income households were significantly more likely to provide them. However, there were no differences in modified formula provision by caregiver race/ethnicity or education level. Therefore, it does not appear that modified formulas disproportionately appeal to different racial/ethnic groups, as has been found with the provision of infant formula in general (4), or that education level affects caregivers' interpretation of product claims.

Of the types of modified formulas examined, sensitive varieties that typically contain less lactose and promise benefits for common infant gastrointestinal issues (e.g., fussiness and gas) were provided most often, followed by organic/non-GMO types. Caregivers provided supplemental and soy-based formulas less often. Caregivers reported purchasing both modified and standard formulas for similar reasons. Agreement with “pediatricians recommend it” and “it has benefits for infants” was highest for both types. These responses are problematic due to the lack of high-quality scientific evidence supporting the benefits of one formula compared with another (18). Furthermore, these responses support concerns raised in other studies that caregivers may infer that their formula has benefits over breast milk given research showing that formula company marketing more often compares their infant formulas to breastfeeding than to other formulas (14). Moreover, as reported in a previous study, the majority of infant caregivers (62%) indicated that common claims about infant formula mean that “Infant formulas can provide nutrition that babies do not get from breast milk” (15).

One significant difference was that caregivers who provided modified vs. standard were more likely to agree that “my pediatrician prescribed it.” Although modified formulas do not require a prescription for purchase, these responses suggest that caregivers may have discussed formula options with their pediatrician who endorsed providing the modified formula in some way. Caregivers who provided modified vs. standard formulas were also more likely to agree that they purchased the product for its natural (e.g., non-GMO and organic) ingredients. This finding suggests that these types of ingredient claims may contribute to caregivers' willingness to pay more for modified formulas.

These findings also support calls for regulations to limit unsubstantiated claims commonly used on formula products (7–9, 13–16), including structure/function claims that link product ingredients (e.g., DHA and low-lactose) to benefits for bodily functions (e.g., brain development and less gas). It appears that approximately one-half of caregivers believed that the added expense of modified formulas is worth the cost. Focus groups with parents of young children have shown that they place significant trust in formula companies and mistakenly assume that claims on product labels have been scientifically proven (21). To address this potentially misleading practice, the FDA issued draft guidance for the industry in 2016 recommending that companies improve the type and quality of scientific evidence companies used to substantiate structure/function claims on infant formula product labels (22). This guidance proposed that product claims on infant formulas should be held to a higher standard than similar claims on food products for the general population. However, the FDA has not finalized this guidance.

Finally, these findings support concerns about the need to restrict all marketing of formulas directly to consumers. In 1981, the WHO adopted the International Code of Marketing of Breastmilk Substitutes (The Code), with the goal of promoting and protecting breastfeeding by addressing aggressive marketing of breast milk substitutes (i.e., formulas) (23). The Code specifies unacceptable formula marketing practices, which include any type of promotion to the general public or through the healthcare system. Notably, the USA remains one of the few countries that has not implemented any articles of the Code (24). Caregivers' high levels of agreement with statements that the formula they provided their child is recommended by pediatricians and benefits their child that indicates the effectiveness of formula company marketing promises at conveying unproven advantages for infants to caregivers and possibly healthcare providers as well.

This study has some limitations. As with all self-reported data, there is the potential for inaccurate or biased responses. Additionally, the cross-sectional study design and sample augments for diverse races/ethnicity do not provide a representative sample of the US population. Moreover, there were 26 (6%) participants who indicated that they had purchased “other” formula products that could not be classified as a standard or modified formula. Approximately one-third of these participants reported serving a private label formula brand, which represents just 5% of all formula sales in the United States (12). Additional research is required to understand participants' responses to some questions. For example, research is needed to assess pediatricians' perceptions of modified formulas, including whether common marketing claims also influence their perceptions of these products, and to better understand when and why they are recommending them to patients. Qualitative research with caregivers who provide both standard and modified formulas would also help better understand how parents make their decisions about the best formula for their child and whether marketing claims lead them to infer that the formula they choose is as good or better for their baby than other formulas and/or breastfeeding.

This study provides additional evidence that modified formulas, with their higher cost and unsubstantiated product claims, are popular with a high proportion of infant caregivers across all demographic groups. It also demonstrates that many caregivers suggest that modified formulas provide benefits for their infant over other formulas. These findings support the need for greater FDA regulation of claims on formula products and government restrictions on formula marketing directed to caregivers.

## Data Availability Statement

The raw data supporting the conclusions of this article will be made available by the authors, without undue reservation.

## Ethics Statement

The studies involving human participants were reviewed and approved by University of Connecticut Institutional Review Board. Written informed consent for participation was not required for this study in accordance with the national legislation and the institutional requirements.

## Author Contributions

MR-P and JH designed the study. MR-P and TR conducted statistical analysis. HG, TR, and JH drafted the manuscript. FF-M and MR-P edited the manuscript. JH and FF-M obtained funding for this research. All authors contributed to the article and approved the submitted version.

## Funding

This study was supported by a grant from the Robert Wood Johnson Foundation, Princeton, NJ. The views expressed in this study do not necessarily reflect the views of the Foundation.

## Conflict of Interest

The authors declare that the research was conducted in the absence of any commercial or financial relationships that could be construed as a potential conflict of interest.

## Publisher's Note

All claims expressed in this article are solely those of the authors and do not necessarily represent those of their affiliated organizations, or those of the publisher, the editors and the reviewers. Any product that may be evaluated in this article, or claim that may be made by its manufacturer, is not guaranteed or endorsed by the publisher.
