# Supplementary material for: Exploring Infant Caregivers' Provision of Modified Formulas: Potential Demographic Differences and Reasons for Provisions
Source: Front Nutr. 2022 May 18;9:867932. doi: 10.3389/fnut.2022.867932 (PMC9172833; doi:10.3389/fnut.2022.867932)
Supplement: Supplementary file 1 [file Table_1.pdf]

Supplementary Materials

List of formula products categorized by formula type

Formula type	Brand	Product name*
Standard formulas		
	Enfamil	Infant
	Enfamil	Newborn
	Enfagrow	Toddler Next Step
	Enfagrow	Toddler Transitions
	Gerber Good Start	Gentle
	Gerber Good Start	Grow
	Nestlé Nido	Fortificada (Fortified)
	Nestlé Nido	Kinder 1+
	Similac	Advance
	Similac	Go & Grow Milk
	Similac	Go & Grow Vanilla
Modified formulas		
- Organic/Non-GMO	Happy Baby	Stage 1 Organic Infant Formula
	Happy Baby	Stage 2 Organic Infant Formula
	Happy Tot	Grow & Shine Organic Toddler Milk
	Plum Organics	Grow Well Organic
	Similac	Advance Non-GMO
	Similac	Go & Grow Non-GMO
	Similac	Organic
- Sensitive	Enfamil	A.R.
	Enfamil	Enspire
	Enfamil	Gentlease
	Enfamil	Reguline
	Enfagrow	Toddler Transitions Gentlease
	Gerber Good Start	Soothe
	Similac	Go & Grow Sensitive
- Supplemental	Enfamil	For Supplementing
	Gerber Good Start	Gentle 2
	Gerber Good Start	Gentle for Supplementing
	Similac	For Supplementation
- Soy	Enfamil	ProSobee
	Enfagrow	Toddler Transitions Soy
	Gerber Good Start	Soy
	Gerber Good Start	Soy 3

*Due to widespread consumer confusion between infant formula and toddler milk (marketed as for children 12-36 months) (15), toddler milk product names were included on the list of products shown to participants for each brand.
